# Live Cell Sorting of Differentiated Primary Human Osteoclasts Allows Generation of Transcriptomic Signature Matrix

**DOI:** 10.21203/rs.3.rs-6157400/v1

**Published:** 2025-04-01

**Authors:** Joshua Lang, Adeline Ding, Erika Henninger, Shannon Reese, Kyle Helzer, Xavier Hazelberg, Cristina Sanchéz de Diego, Sheena Kerr, Nan Sethakorn, Matthew Bootsma, Shuang Zhao, David Beebe

## Abstract

Osteoclasts are specialized cells that degrade the bone matrix to create space for bone regeneration. During tumorigenesis, cancer cells metastasize to bone by disrupting bone’s natural remodeling cycle. However, the mechanisms underlying critical bone-tumor interactions are poorly understood due to challenges in isolating osteoclasts from human bone. Thus, the conventional method to obtain osteoclasts for in vitro studies is via the differentiation of peripheral blood monocytes, which results in mixed cultures containing progenitor cells and osteoclasts of varying maturity and nuclearity. Presently, we hypothesized that the transcriptomic signatures of mature, multinucleated osteoclasts are distinct from osteoclasts with fewer nuclei. We established a live cell biomarker expression-based sorting protocol to allow purification of mature osteoclasts while maintaining viability and function. We observed that mature, multinucleated osteoclasts were transcriptomically distinct from those with fewer nuclei and that mature osteoclasts showed higher expression of genes that are associated with osteoclast fusion and function.

## INTRODUCTION

Osteoclasts are a critical component of the bone microenvironment that work in tandem with osteoblasts to maintain healthy bone structure and homeostasis[1–3]. Osteoclasts are myeloid lineage cells that break down and absorb mineralized bone matrix to allow tissue repair and create space for osteoblast-driven bone regeneration. Osteoblasts are specialized bone cells that function reciprocally to osteoclasts by building new bone tissue. Osteoclast-mediated bone matrix degradation induces secretion of transforming growth factor-β (TGF-β), bone morphogenic protein (BMP), collagenases, and other proteases. TGF-β promotes osteoclast differentiation while BMPs promote osteoblast differentiation[4]. During tumor pathogenesis, cancer cells metastasize to bone and then expand by disrupting the bone's natural remodeling cycle. In concert with stroma-secreted paracrine signals, the invasive tumor cells ultimately adapt the bone's complex stromal networks to degrade the bone scaffold and condition the microenvironment to establish metastatic niches[2,3,5–12]. The resulting bone tumor microenvironment has been also associated with therapeutic resistance[13].

However, the mechanisms behind these critical bone-tumor interactions that drive the metastatic cascade and promote resistance are poorly understood partly due to the lack of available translationally relevant models to study human bone tissue biology and tumor biology in the context of the osseous microenvironment. Notably, technical limitations in isolating intact human osteoclasts from primary tissue have limited both the investigation and use of osteoclasts in subsequent mechanistic and functional studies. In general, isolation and *ex vivo* maintenance of primary osteoclasts from bone is widely regarded as technically challenging and largely limited to animal models[14]. Animal model-based *ex vivo* tissue processing techniques include physical and biochemical fragmentation of bone that results in loss of viable cellular content, and therefore these methods have limited utility in cellular molecular discovery[14]. Of the human *ex vivo* models of bone that exist, they largely consist of explant cultures of trabecular bone and have been associated with significant loss in osteoclast function and viability[15,16]. Thus, the conventional method to obtain osteoclasts for *in vitro* studies is established through *in vitro* differentiation of peripheral blood monocytes in the context of Macrophage Colony Stimulating Factor (M-CSF) and Receptor Activator of Nuclear Factor Kappa B Ligand (RANKL), which stimulate fusion of osteoclast progenitors to create multinucleated cells.

Early osteoclast differentiation is driven by binding of osteoblast-secreted M-CSF to its receptor, CSF1R (colony stimulating growth factor 1 receptor), on osteoclast precursor cells[2,17,18]. Importantly, M-CSF/CSF1R binding triggers expression of RANK on osteoclast precursors, which binds to osteoblast-secreted RANKL to induce a MAPK (mitogen-activated protein kinase) and NF-κB (Nuclear factor kappa-light-chain-enhancer of activated B cells) signaling cascade to activate NFATc1 (Nuclear factor of activated T-cells, cytoplasmic 1), the key regulator of osteoclast differentiation[2,17–19]. Once mature, osteoclasts adhere to the bone scaffold via αVβ3 integrin (CD51/61), which is critical for bone resorption[20–22]. CD51 and CD61 form a glycoprotein heterodimer and mediate adhesion to extracellular matrix filaments including fibrinogen, fibronectin, vitronectin, and thrombospondin. Within the multi-step osteoclast differentiation process, the role and functionality of precursors versus mature osteoclasts are poorly understood due to limited data available for molecular investigation of specific human bone cell types, including osteoclasts. A recent study examined transcriptional changes during the human osteoclast differentiation process by performing RNA sequencing at different stages, including starter cultures of peripheral blood mononuclear cells (PBMCs)[23]. Osteoclasts, however, were not purified from their mixed cultures prior to sequencing. Thus, the extent to which the study's findings are specific to mature osteoclasts, as opposed to less differentiated precursor cell types, is unclear.

Additionally, the overall efficiency of *in vitro* human monocyte-derived osteoclast differentiation protocols is limited and variable, as reported in several published protocols[24,25]. In our study, we hypothesized that the transcriptomic signatures of mature, multinucleated osteoclasts are distinct from less differentiated progenitors with fewer nuclei. To investigate this, we developed an efficient method of purifying viable osteoclasts from mixed monocyte/macrophage/osteoclast populations to allow osteoclast-specific molecular analysis and subsequent future mechanistic assays.

We have established a live cell sorting protocol for purification of mature osteoclasts by utilizing biomarker expression-based identification of differentiated human osteoclasts while maintaining viability and function. To distinguish mature osteoclasts from less differentiated precursors, we developed a gating strategy for cell sorting based on surface expression of CD51/61 (αVβ3 integrin) and RANK, and nuclearity. To validate osteoclast enrichment, we extracted RNA from baseline specimens and matched purified osteoclast subsets to assess osteoclast marker gene expression by qPCR analysis and bulk RNA sequencing. We observed that mature, multinucleated osteoclasts were transcriptomically distinct from their progenitors with 1–2 nuclei and that, in comparison with these less nucleated osteoclasts, mature osteoclasts showed higher expression of genes that were associated with osteoclast fusion and function.

## METHODS

### Human Blood Specimens

Blood samples were collected from biologically male patient donors with prostate cancer receiving treatment at University of Wisconsin Carbone Cancer Center (UWCCC) after receiving written informed consent under a protocol approved by the Institutional Review Boards at the University of Wisconsin-Madison (#2014 – 1214). Research has been performed in accordance with the Declaration of Helsinki. Blood specimens were collected in vacutainer tubes (BD Biosciences, Franklin Lake, NJ, USA #367863) with EDTA anticoagulant. Whole blood was diluted 1:1 with Hank's balanced salt solution (HBSS, Corning, Thermo Fisher Scientific, Waltham, MA USA #21022CM) followed by underlaying with 10 ml of Ficoll-Paque PLUS (Cytiva, Fisher Scientific, Thermo Fisher Scientific, Waltham, MA USA Cat# 45-001-750) for gradient centrifugation and isolation of the PBMC fraction from the buffy coat.

### Osteoclast Differentiation Culture

$CD14^+$ monocytes were enriched from PBMCs using anti-CD14 magnetic beads (Miltenyi Biotec Inc., Bergisch Gladbach, North Rhine-Westphalia, Germany #130-050-201) and LS MACS columns (Miltenyi Biotec Inc., Bergisch Gladbach, North Rhine-Westphalia, Germany #130-050-201) following manufacturer's protocol, then seeded into tissue culture treated 24-well plates at 1.5 to 2 million cells per well in macrophage differentiation medium, including Dulbecco's Modified Eagle Medium (Corning, Corning, Thermo Fisher Scientific, Waltham, MA USA # MT10017CV) supplemented with 10% fetal bovine serum by volume (R&D Systems, Minneapolis, MN, USA #S11550H), 1% penicillin/streptomycin (Hyclone, Cytiva, Marlborough, MA USA #SV30010) and 25 ng/mL M-CSF (R&D Systems, Minneapolis, MN, USA #216-MC-025/CF). Cells were incubated at 37° C with 5% $CO_2$ for 48 hours and then an equal volume of macrophage differentiation medium was added to each well. After observation of adhesion of cells to the plate (at around 4–5 days), macrophage differentiation media was exchanged for osteoclast differentiation medium (Dulbecco's Modified Eagle Medium supplemented with 10% fetal bovine serum by volume, 1% penicillin/streptomycin with 25 ng/mL M-CSF and 50 ng/mL RANK-L (PeproTech, Thermo Fisher Scientific, Waltham, MA USA #310-01) and cultured for 8–10 days.

### Cell sorting

Differentiated osteoclast cultures were harvested using 500 µl Accutase (Innovative Cell Technologies, Thermo Fisher Scientific, Waltham, MA USA # NC9839010). Specifically, after 20 minutes of incubation with Accutase, another 500 µl Accutase was added. Cells were then gently dissociated from the plates using a P1000 pipet and transferred to magnetic-activated cell sorting (MACS) buffer-coated tubes (STEMCELL Technologies, Vancouver, Canada #38007) followed by centrifugation for 5 minutes at 300 RCF. The supernatant was discarded and cells in each tube were resuspended in 1 mL of fresh MACS

buffer, pooled into one tube, counted, and centrifuged again for 5 minutes at 300 RCF. The supernatant was discarded and cells were resuspended in 100 µl of MACS buffer.

5% of cells were transferred to a new MACS-coated tube for each FMO control to establish gating thresholds for RANK and CD51/61 binding and 5% of cells were used for an unstained control. Additionally, 2.5% of total harvested cells were transferred into 100 µl of MACS buffer in a 1.7 mL microcentrifuge to generate single-color compensation controls including Hoechst 33342 (Hoechst; Thermo Fisher Scientific, Waltham, MA USA #62249) staining. To generate a compensation control for live/dead stain, 2.5% of cells were transferred into 100 µl of MACS buffer in a 1.7 mL microcentrifuge tube and heat-killed in a 65° C water bath for 15 minutes before staining with 1 µl of 780 Ghost Dye (Tonbo Biosciences, Cytek, San Diego, CA, USA #13-0865-T100). The remaining 75% of cells were processed for cell sorting. For the full stain, 10 µl BD Pharmingen FITC Mouse Anti-Human CD51/CD61 (BD Biosciences, Franklin Lakes, NJ USA #555505, RRID: AB_2129630), 5 µl Invitrogen PE RANK Monoclonal Antibody (9A725) (Invitrogen, Thermo Fisher Scientific, Waltham, MA USA #MA1-41015 RRID: AB_1087023), 1 µl BioLegend Alexa Fluor® 647 anti-human CD14 (BioLegend, San Diego, CA USA #325612 RRID: AB_830685), and 1 µl Cytek® Tonbo™ Ghost Dye™ Red 780 were added. Cells were incubated for 30 minutes at RT in 100 µl of final volume in MACS buffer, protected from light, followed by washing in 1 mL of MACS buffer and centrifugation for 5 minutes at 300 RCF. Cells were resuspended in 400 µl MACS buffer and a 1:100 dilution of 10 mg/mL Hoechst dye was added to each tube. Single stain compensation controls were prepared with UltraComp eBeads (Invitrogen, Thermo Fisher Scientific, Waltham, MA USA #01-2222-41). Compensation controls for Hoechst and Ghost Dye 780 single stain were prepared with cells from the current differentiated osteoclast cultures sorted on the same day. After washing with MACS buffer and before sorting, cell suspensions were filtered through a 40 µm sieve cap.

Samples were sorted on a BD FACS Aria II SORP Cell Sorter in the University of Wisconsin Carbone Cancer Center Flow Cytometry Laboratory using a 130 µm nozzle and 14 psi pressure. Cytometer Setup and Testing & Quality Control was performed and documented daily by core services. Prior to sorting, the fluidics line and flow cell were flushed thoroughly with RNase Away (Thermo Fisher Scientific, Waltham, MA USA #7005-11). Basic gating strategy included gating on intact cellular events projected on the Forward Scatter and Side Scatter (Cell gate), followed by dead cell exclusion (Live gate). Gating thresholds for biomarkers including CD51/61 and RANK were established using fluorescent minus one (FMO) strategy for each biomarker (Supplementary Fig. 1A, B). Osteoclasts were sorted in 'Purity' precision mode with Purity Mask, Yield Mask and Phase Mask set at 32, 32, and 0, respectively. Events in the Cells/Live gate were projected on the CD51/61 versus CD14 expression spectrum in dot plots. The $CD51/61^{+}$ /$CD14^{low}$ events were then projected on the RANK expression versus FSC-Area. The $CD51/61^{+}$/$RANK^{+}$ double positive cells were then rendered into two subsets for sorting including High Hoechst signal to capture mature osteoclast cells with multiple, > 2 nuclei, visualized as projected on a histogram and Low Hoechst cells with 2 or fewer nuclei.

### Subculture of Sorted Cell Populations

A fraction of live cells was assessed for post-sort viability with AO/PI double staining on a Nexcelom Cellometer (Revvity, Waltham, MA, USA) following manufacturer's protocol. Cells were then seeded in a tissue culture-treated 96-well flat bottom plate at ~ 10,000 cells per well and maintained in osteoclast differentiation media. Cell adhesion, morphology, and culture quality were assessed by daily observation for up to 7 days and images were captured with brightfield microscopy with a 10x objective using Nikon Eclipse Ti-E with an ORCA-Flash 4.0 V2 Digital CMOS camera (Hamamatsu) and NIS-Elements AR Microscope Imaging Software (RRID:SCR_014329, Nikon Instruments).

For functional assessment, 0.1 mg Sera-Mag beads (Thermo Fisher Scientific, Waltham, MA USA # 21152104011150) were washed in osteoclast differentiation media, resuspended into single beads and were evenly distributed on the adherent monolayers of sorted osteoclasts sub-cultured in 96-well plates. Cells were maintained at 37C with 5% $CO_2$ and monitored daily.

### RNA extraction

Immediately prior to cell sorting, 5% of each unsorted culture was snap frozen in RLT Plus lysis buffer RNeasy Plus Micro Kit (Qiagen, Hilden, North Rhine-Westphalia, Germany #74034) with β-ME and stored at -80°C, to serve as the "bulk" presort sample. Live cells were sorted directly into RLT Plus lysis buffer with 1:100 β-ME (Qiagen RNeasy Micro Kit) and snap frozen and stored at − 80°C until processing, following manufacturer's protocol. For all samples, cells were lysed in RLT Plus buffer with 1:100 β-ME added. Samples were thoroughly vortexed and run through QIAshredder (Qiagen, Hilden, North Rhine-Westphalia, Germany #79656) for homogenization. Total RNA was then isolated from samples using the RNeasy Plus Micro Kit. RNA quality and quantity was assessed using both the Qubit RNA High Sensitivity Assay Kit (Invitrogen, Thermo Fisher Scientific, Waltham, MA USA #Q32852) on a Qubit Fluorometer (Thermo Fisher Scientific, Waltham, MA USA #Q33238, RRID:SCR_018095) and the Agilent High Sensitivity RNA ScreenTape assay (Agilent Technologies, Santa Clara, CA USA #5067–5579 #5067–5580 #5067–5591) on the Agilent Tapestation 4200 (Agilent Technologies, Santa Clara, CA USA #G2991BA, RRID:SCR_018435).

## Tartrate Resistant Acid Phosphatase (TRAP) staining

The Leukocyte Acid Phosphatase (TRAP) Kit (Sigma-Aldrich, Millipore Sigma, Burlington, MA, USA #387A-1KT) was used following the manufacturer's instructions. After fixation of cells with BD Cytofix Fixation Buffer (BD Biosciences, Franklin Lakes, NJ USA #554655, RRID: AB_2869005), a one-to-one solution (2% final volume) of Fast Garnet GBC Base Solution and Sodium Nitrite Solution was mixed with Naphthol AS-BI Phosphate Solution (1% final volume), Acetate Solution (4% final volume), Tartrate Solution (2% final volume), and prewarmed deionized water (90% final volume). Cells were incubated for 1 hour at 37°C protected from light. After 1 hour, cells were rinsed thoroughly in deionized water, then counterstained for 2 minutes in Hematoxylin Solution.

### Quantitative polymerase chain reaction (qPCR)

Extracted RNA was reverse transcribed using the High-Capacity RNA-to-cDNA™ Kit (Applied Biosystems, Thermo Fisher Scientific, Waltham, MA USA #4387406), according to manufacturer's directions. The RT reaction was then amplified for 14 cycles using TaqMan™ PreAmp Master Mix (cDNA pre-amplification for qPCR) (Applied Biosystems, Thermo Fisher Scientific, Waltham, MA USA #4391128) according to manufacturer's directions. For TaqMan® assays, 10 µL iTaq Universal Probes Supermix (Bio-Rad, Hercules, CA, U.S.A #1725134) and 4 µL nuclease free (NF) water was combined per test to make Master Mix 1 (MM1). 14 µl MM1 was combined with 1 µl Taqman primer per test to make Master Mix 2 (MM2). Finally, 15 µl of MM2 was combined with 5 µl pre-amplified cDNA per well of the PCR plate. Reactions were amplified for 45 cycles using a CFX Connect® Real-Time PCR System (Bio-Rad, Hercules, CA, U.S.A). Specific primers are listed in Table 1.

### cDNA and library prep cDNA was synthesized from RNA using the SMART-Seq® v4 Ultra® Low Input RNA Kit for Sequencing kit (Takara Bio Inc., Kusatsu, Shiga, Japan #634773). After quantification of cDNA using the High Sensitivity D5000 ScreenTape assay (Agilent Technologies, Santa Clara, CA USA #5067–5592 #5067–5593) and High Sensitivity dsDNA Quantitation Qubit assay (Invitrogen, Thermo Fisher Scientific, Waltham, MA USA #Q32851), library preparation was performed using the Nextera XT DNA Library Preparation Kit (Illumina, San Diego, CA, USA #15032354). DNA libraries were quantified using the Qubit 1x HS dsDNA kit and the Agilent High Sensitivity D1000 DNA kit (Agilent Technologies, Santa Clara, CA USA #5067–5585 #5067–5584) and stored at -20° C.

## Bulk RNA sequencing

Paired end sequencing of pooled libraries was performed at the University of Wisconsin Biotechnology Center Gene Expression Center on a NovaSeq 6000 (Illumina) platform (RRID:SCR_016387) with 150 bp read length.

## RNAseq data pre-processing

Samples were sequenced on an Illumina NovaSeq 6000 platform (RRID:SCR_024569) to a depth of [insert depth requested] reads with 150 bp paired-end sequencing (2x150 bp) per read. Reads were aligned using STAR1 (v2.7.6a) to an index built from the hg38 genome (GRCh38.p13) using NCBI Homo sapiens updated annotation release 109.20200815. The STAR alignment was performed using default parameters except for the following: --runThreadN 20; --quantMode GeneCounts; --outSAMtype BAM SortedByCoordinate. Sorted BAM files were then converted to read counts using featureCounts2 in the SubRead package (Subread v2.0.1) using the annotation release stated above along with the following parameters: -g "gene" -T 64 -B -p -Q 10 -s 0. Raw read counts were then converted to transcripts per million (TPM) using a custom R script (R v4.3.0).[26,27]

### Differential gene expression analysis

Differential expression analysis was performed using the DESeq2 package in R. The differential expression analyses controlled for cell fraction (design = ~ fraction_ID), where fractions were RNAseq profiles of either "Bulk", unsorted differentiated osteoclasts, "Low", sorted CD51/61$^+$ RANK$^+$ osteoclasts with low Hoechst signal indicating single nuclei per cell, "Med", sorted CD51/61$^+$ RANK$^+$ osteoclasts with Hoechst signal indicating two to three nuclei per cell, and "OC", CD51/61$^+$ RANK$^+$ osteoclasts with high Hoechst signal indicating multiple nuclei per cell and mature osteoclast phenotype. Genes with log2FoldChange great than or less than 2 and p-value less than 0.05 were considered differentially expressed gene (DEG).

### Visualization of differential expression

Differential expression of genes was visualized in R using the ggplot package.

### Gene set enrichment analysis (GSEA)

GSEA was conducted in R using the fgsea package and Gene Ontology (GO) Biological Processes (BP) human gene set Osteoclast Differentiation (GO:0045670) and WikiPathways human gene set Osteoclast Signaling (WP12).

## RESULTS

### Primary human osteoclast differentiation from CD14$^+$ peripheral monocytes

PBMCs were isolated from whole blood from eight patient donors with metastatic prostate cancer. Following CD14$^+$ monocyte enrichment, cells were maintained in macrophage differentiation medium followed by osteoclast differentiation with M-CSF and RANK-L (Fig. 1A). Cultures were observed daily and assessed for morphological changes including cellular fusion. We found that multinucleated cells started forming between 4 and 5 days in most donor cultures and the length of differentiation was typically occurring between days 8–10 (Fig. 1B). To qualitatively assess the differentiation phenotype and cellular morphology, we performed histochemical staining of the differentiated cultures to look for the formation of tartrate-resistant acid phosphatase (TRAP) granules (Fig. 1B). We found that TRAP$^+$ granules were present in the multi-nucleated cells suggesting that these cells represented mature osteoclast phenotype. Next, we assessed surface expression of CD51/61, a marker of osteoclast adhesion, using fluorescent microscopy (Fig. 1C). We observed that differentiated multi-nucleated osteoclasts were distinct from mononucleated cells as CD51/61 surface binding associated with the multi-nucleated fraction. Meanwhile, the majority of the mononuclear subset in differentiated osteoclast cultures remained negative for CD51/61 expression (Fig. 1C). In accordance with previously established osteoclast differentiation protocols, at the end of the differentiation period, we have observed that cultures contained a mixed population of multinucleated mature osteoclasts, di- or tri-nucleated osteoclasts, and macrophage/monocyte osteoclast precursors with considerable inter-donor variability.

## Live cell sorting of mature, viable osteoclasts from two-dimensional adhesion cultures

To allow gentle harvest of mature osteoclasts from the differentiated monolayers, cells were detached using Accutase and mild physical agitation. After dissociation with Accutase, cell viability was assessed using simultaneous propidium iodide and acridine orange (AOPI) staining. Average cell viability before cell sorting was 86.36% ± 10.53 (Supplementary Fig. 1C).

To enrich mature osteoclasts, we leveraged biomarker expression patterns to identify this specific subset in our heterogenous differentiated osteoclast cultures. To achieve this, we sequentially gated on double-positive subsets co-expressing CD51/61 and RANK surface protein within the $CD14^{-}$ subset. Our gating strategy is shown in Fig. 2A. Our gating thresholds were validated by the Fluorescent Minus One (FMO) strategy described in Methods and shown in Fig. 2A, B. Specifically, events in the Cells/Live gate were projected on the CD51/61 versus CD14 expression spectrum in dot plots. The $CD51/61^{+}/CD14^{low}$ events were then projected on the RANK expression spectrum versus FSC-Area. The $CD51/61^{+}/CD14^{-}/RANK^{+}$ cells were rendered into two subsets for sorting visualization and gates were established on the Hoechst spectrum. We sorted ‘Low Hoechst or Low’ cells to enrich low-nuclei count events (≤ 2 nuclei) and ‘High Hoechst or OC’ events with multiple nuclei (> 2 nuclei) to capture a distinct subset of more mature multinucleated osteoclasts. Subsequently, the Low Hoechst cells will be referred to as “Low” and the High Hoechst cells will be referred to as “OC”.

To assess post-sort viability, we established live nucleated cell counts with AO/PI staining on a Nexcelom Cellometer. The average viability was 96.48% ± 5.045 and 100% ± 0.00, in Low Hoechst and High Hoechst cells, respectively (Supplementary Fig. 1D). Viability was further assessed with a Calcein AM-ethidium homodimer viability assay for live cell content and ethidium homodimer staining to capture cells death, acquired by fluorescent microscopy. The sorted osteoclasts, both Low and OC, displayed strong Calcein AM uptake and minimal ethidium homodimer signal, consistent with the cell counter data (Fig. 3A). A sample of each sorted Low Hoechst and High Hoechst fraction was washed into osteoclast differentiation media followed by plating into 96-well cell-culture plates at around 10,000 cells per well and cells were sub-cultured overnight to allow for adhesion. By Day 3 of sub-culture, the sorted Low and OC cells were adherent to the tissue culture plates and were forming membrane extensions towards other osteoclasts present in culture, demonstrating viability post-sorting. (Fig. 3B). As monocyte and macrophage-derivatives, it is expected that functional osteoclasts retain the phagocytic functions of their progenitors to enable bone resorption mechanisms[28]. A single cell suspension of ferromagnetic particles was added to subculture wells to allow even distribution around the cellular monolayer. Within 24 hours, we observed the formation of membrane extensions towards magnetic bead clusters and dead cells

(identified by cell morphology) in Low and OC cultures (Fig. 3C). Furthermore, with daily observation of the same areas and specific cells, we have captured events of adherent osteoclasts engulfing beads into the main cellular body captured by bright-field imaging. As shown in Fig. 3C, the farthest left osteoclast can be seen forming extensions towards a clump of magnetic beads on Day 3 of subculture. By Day 4 of follow-up, that same clump of beads was visible in the main body of the same osteoclast. Over time, the osteoclasts gradually cleared their surrounding area of magnetic beads. This phagocytic activity was observed up to 7 days in culture after live cell sorting, suggesting robust post-sort viability and maintained phagocytic function of differentiated osteoclasts. We also observed that the two sorted subsets, "Low Hoechst" and "High Hoechst" showed distinct cellular features. As expected, the "Low Hoechst" cells represented morphologically less mature osteoclasts containing few nuclei per cell, while the "High Hoechst" subset represented cells that contained multiple nuclei. The "High Hoechst" subset was subsequently referred to as "osteoclasts" or "OC" in molecular analysis. The majority of sorted cells in both subsets showed robust post-sort viability and membrane integrity as they were able to adhere to the flasks and developed long membrane extensions. We observed more membrane extensions on the "High Hoechst" cell subset.

## qPCR analysis confirms enrichment of osteoclast genes in purified cell fractions

To assess the efficiency of live cell sorting enrichment in our purified osteoclast subset, we isolated mRNA from differentiated osteoclast culture specimens prior to cell sorting (Presort) and

High Hoechst (OC) cells. For rapid assessment of molecular signatures of the target osteoclast population, we performed qPCR analysis of relative expression of osteoclast markers genes including Acid Phosphatase 5, Tartrate Resistant (*ACP5*), cathepsin K (*CTSK*), matrix metalloproteinase 9 (*MMP9*) and macrophage marker CD163 (*CD163*). Compared to Presort, OC had greater relative expression of *ACP5*, *MMP9*, and *CTSK*, while Presort had higher relative expression of *CD163* (Supplementary Fig. 1E). Thus, the gain of osteoclast marker gene expression and loss of macrophage gene expression confirmed enrichment of osteoclasts in the OC subfractions and supported that our live cell sorting approach utilizing CD51/61, RANK and nuclear content as biomarkers enriched the desired target cell populations from the heterogenous osteoclast differentiation cultures. A subset of patients, however, displayed mixed patterns of relative expression of *CTSK*, *ACP5*, *MMP9*, and *CD163*, suggesting inter-patient heterogeneity and highlighting the need for RNA sequencing for broader examination and identification of osteoclast target genes.

## RNAseq analysis identifies unique transcriptomic patterns of mature monocyte-derived primary osteoclasts

In order to further assess the molecular patterns in our purified, sorted osteoclast isolates and investigate the transcriptomic variation between more or less nucleated osteoclasts, we performed bulk RNA sequencing on matched CD14 ($CD14^+$ monocyte starter culture) precursors, Presort, and sorted Low (Low Hoechst) and OC (High Hoechst) osteoclast subsets (Fig. 4A). Gene set enrichment analysis

(GSEA) was performed using two osteoclast-specific gene sets accessed from the Molecular Signatures Database (MSigDB): 1) Gene Ontology (GO) Biological Processes (BP) human gene set Osteoclast Differentiation and 2) WikiPathways human gene set Osteoclast Signaling. As expected, comparing CD14 to OC samples, GSEA showed significant enrichment for both osteoclast signaling and differentiation in the OC samples, thus confirming successful differentiation of osteoclasts at a transcriptomic level (Fig. 4B). Clustering via UMAP

dimensionality reduction showed that the CD14 fraction was distinct from Presort, Low, and OC specimens (Fig. 4C). Interestingly, although the Presort cell fractions were a heterogenous mixture of immune osteoclast precursors, Low, and OC osteoclasts, the Presort samples across donors generally grouped together. Though small groups in Low and OC samples also emerged, more heterogeneity was observed between those two sample types (Fig. 4C). This was consistent with the phenotypic and functional validation of osteoclast differentiation (Fig. 3C, E). Accordingly, analysis of expression of a larger panel of osteoclast marker genes showed strong expression in Presort, Low, and OC samples and low expression in the CD14 samples (Fig. 4D). Specifically, DESeq2 analysis comparing OC samples to the $CD14^+$ monocyte progenitor samples observed significant enrichment (log2FoldChange > 2, alpha = 0.05) for osteoclast marker genes in OC including *MMP9*, *ACP5*, *CTSK*, sialoprotein 2/osteopontin (*SPP1*), TNF receptor superfamily member 11a (T*NFRS11a* or *RANK*), sialic acid binding Ig like lectin 15 (*SIGLEC15*), osteoclast stimulatory transmembrane protein (*OCSTAMP*), and *DCSTAMP* was observed. Conversely, differential expression of monocyte markers such as *CD14* and cell surface transmembrane glycoprotein CD200 receptor 1 (*CD200R1*) was significantly increased in the $CD14^+$ fraction compared to OC (Fig. 4E).

We next performed DESeq2 analysis to compare sorted mature OC samples versus matched Presort specimens and found that OCSTAMP was still significantly upregulated in OC compared to Presort (Fig. 4F). OCSTAMP is a significant regulator cell-to-cell fusion representing a critical step in osteoclast differentiation to allow formation of mature, multinucleated osteoclasts (PMID: 22337159). Additionally, other genes that have been associated with osteoclastogenesis and bone turnover, including tenascin-N/W (*TNN*), glucagon-like peptide 2 (*GLPR2*), TEK receptor tyrosine kinase (TEK/TIE2) were also significantly upregulated (log2FoldChange > 2, alpha = 0.05) in OC compared to Presort (Fig. 4F) [29–33]. In general, although we observed fewer total osteoclast marker genes that associated with significant differential expression in 'OC vs Presort', compared to 'OC vs $CD14^+$', several osteoclast genes such as *CALCR, CTSK, ACP5, SIGLEC15* had a higher fold expression in OC compared to Presort. Together, these data indicate distinct transcriptomic signatures captured between enriched mature osteoclasts and bulk presort culture specimens, supporting the efficacy of our biomarker-based live cell sorting protocol to generate purified analytes for subsequent transcriptomic analysis of osteoclast-specific signatures.

## Multinucleated $CD51/61^+/RANK^+/CD14^-$ osteoclasts are transcriptomically distinct from $CD51/61^+/RANK^+/CD14^-$

### osteoclasts with fewer nuclei

Osteoclast differentiation is a multistep process *in vitro*, where starter cultures of circulating immature CD14$^{+}$ monocytes are first differentiated into M2-polarized macrophages with the addition of M-CSF and then further differentiation and fusion into osteoclasts is induced by the addition of RANK (Fig. 1A). To assess the transcriptomic profiles of these various differentiation phases, we first compared CD14 specimens with differentiated, sorted Low or OC subsets.

We examined the expression of monocyte and M2-polarized macrophage genes across samples. As expected, we observed enrichment of monocyte marker genes in the CD14 samples, and moderate expression of some M2 macrophage marker genes (Fig. 5A). Conversely, in both Low and OC, the expression pattern for monocytes decreased relative to CD14 while the expression of M2 macrophage genes increased, reflecting the directionality of differentiation from monocytes to M2 macrophages to osteoclasts (Fig. 5A, Fig. 4A). The Low samples, however, had a stronger M2 macrophage signature than OC, suggesting that osteoclasts downregulate the expression of some M2 lineage markers as they mature and fuse to gain more nuclei. Consistent with the DESeq2 findings that *OCSTAMP*, a key modulator of cell-to-cell fusion, is upregulated in OC fractions, greater expression of the GOBP gene set Regulation of Syncytium Formation by Plasma Membrane Fusion was observed in 4 OC fractions relative to their matched Low fractions (Fig. 5B). Expression patterns were mixed for the remainder of the matched samples (Supplementary Fig. 2B). Additional genes previously implicated in cell-to-cell fusion in monocyte/macrophage lineage, such as Rap guanine nucleotide exchange factor 3 (*RAPGEF3*), *CD53*, Triggering receptor expressed on myeloid cells 2 (*TREM2*), and tumor necrosis factor superfamily member 14 (*TNFSF14*), showed the strongest enrichment in OC compared to Low (Fig. 5B)[34–37]. Similarly, for the GOBP Osteoclast Fusion gene set, T Cell Leukemia Translocation Altered (*TCTA*) expression was enriched in OC for Pairs 3, 4, 5 and *DCSTAMP* was enriched in OC for Pairs 1, 2 (Fig. 5C). OC fractions showed enrichment for *CD81*, *CD109* and SH3 and PX domains 2A (*SH3PXD2A*) in several other matched pairs. Finally, matched pairs of Low and OC from two donors showed strong enrichment for the GOBP Nucleus Organization gene set in OC, while the remaining matched pairs from other donors showed more variable expression patterns across most genes in the gene sets (Fig. 5D, Supplementary Fig. 3A, B). Together, our findings demonstrated differential enrichment for genes associated with cell-to-cell fusion and nucleus organization in multinucleated osteoclasts compared to less mature and less differentiated mononucleated osteoclasts, highlighting the differences in function and maturity between sorted Low Hoechst and High Hoechst populations in *in vitro* osteoclast cultures. Furthermore, as expected, the data also illustrated patient donor-to-donor differences in primary monocyte-derived osteoclast signatures.

## DISCUSSION

Osteoclasts provide essential function to bone remodeling in both health and disease. However, the knowledge base for human osteoclast biology and their role in pathogenesis is largely limited by lack of access to pure osteoclast isolates for cellular molecular analysis. In this study, we present a primary osteoclast live cell sorting protocol to purify mature, viable, and functional osteoclasts that also allows isolation of osteoclast specific RNA from bulk *in vitro* monocyte-derived cultures by leveraging CD51/61, RANK, and CD14 expression patterns and [20–2220–22]. Additionally, RANK has been established as another important biomarker for mature osteoclasts, representing a key receptor in osteoclast differentiation and osteoclast activation signaling cascades. Thus, utilizing CD51/61 and RANK expression patterns provided a potent approach to develop a dependable gating strategy to identify mature osteoclasts in our live cell sorting protocol. The subsequent bulk RNAseq datasets generated from the sorted osteoclast subsets and bulk presort samples, with direct comparison to matched $CD14^+$ monocyte precursor samples, addressed a long-time need for transcriptomic data on pure primary human osteoclasts.

The bone microenvironment is a complex cellular niche that includes multiple distinct bone cell types including osteoclasts, osteoblasts, osteocytes among others. However, our understanding of the complex bone tissue networks and function has been hindered by the lack of available molecular data sets for these specific cell types and limit investigation into the role of each different bone cell type in bone homeostasis and in skeletal pathogenesis. Isolation and purification of intact bone cells are notoriously difficult. In recent years, there have been a couple of published protocols for live cell sorting of primary human osteoclasts. The first reported method published in 2018 by Madel et al. was based on a gating strategy for nuclear Hoechst enrichment, separating fused multi-nucleated cells with more than three nuclei from mononuclear cells. However, unlike with murine osteoclasts, the authors reported limited efficacy in the context of human osteoclasts and showed reduced viability after Hoechst staining[38]. In 2022, Hulley et al. reported improved preservation of human CD14 monocyte-derived osteoclast viability and function when sorting osteoclasts cultured in 3D collagen matrix[39]. That protocol utilized a CD9 and CD51/61 expression-based gating strategy. However, similarly to the 2018 report, authors detected toxicity that associated with Hoechst 33342 staining[38,39]. Additionally, while efficacy for live cell sorting of human osteoclasts has been improved in 3D cultures, sorting osteoclasts cultivated in 2D monolayers remained limited[39]. As fusion-driven multinucleation is a key characteristic of late phase osteoclast differentiation, this feature serves as a valuable biomarker for isolation of more mature osteoclasts when integrated with canonical cell surface marker expression-based selection[40,41]. Here, we successfully incorporated Hoechst 33342 into our sorting protocol for monolayer-cultured osteoclasts by limiting labeling to a brief, 10 minutes process immediately before sorting, thus allowing for the isolation of multinucleated or mononucleated osteoclasts with maintenance of high post-sort viability and function (Fig. 3). Similar to previous reports, in our study, $CD51/61^+$ / $RANK^+$ co-expressing cells segregated into two distinct subpopulations on the Hoechst enrichment spectrum: Low Hoechst and High Hoechst. In accordance with phenotypic observations, we expected that “Low Hoechst” events represented less differentiated osteoclasts (≤ 2 nuclei) while “High Hoechst” subsets represented fused, multi-nucleated (2+), more mature subsets of differentiated osteoclasts. Importantly, we found that the ‘High Hoechst’ osteoclasts associated with distinct transcriptomic signatures when compared to the ‘Low Hoechst’ osteoclasts and enrichment for genes associated with fusion and nuclear organization expected to be present in more differentiated osteoclasts (Fig. 5). Additionally, our observation that macrophage and monocyte markers are downregulated in mature, multinucleated osteoclasts (Fig. 5A) is consistent with a previous study that observed decreased monocyte-macrophage gene expression concordant with the directionality of osteoclast differentiation and gain of osteoclast marker gene expression[23]. Indeed, a body of literature has described multinucleation as the latest stage of the osteoclast differentiation process and a hallmark of osteoclast maturity[40]. Consistent with our data, osteoclasts with fewer nuclei have been reported to retain markers of osteoclast identity but reduced resorptive activity[40–47]. Interestingly, increased multinuclearity in osteoclasts was previously correlated with decreased sensitivity to the bisphosphonate zoledronic acid (ZA), a drug commonly used to manage bone metastases from solid tumor cancers and other bone diseases[48]. The same study also noted patient-to-patient heterogeneity in ZA efficacy on osteoclasts.

Inter-donor heterogeneity for osteoclast function has been well established for primary human osteoclast cultures[40–42]. Additionally, donor-specific gene expression patterns have been noted in other studies of primary human osteoclasts[23]. We observed this inter-patient variability when examining genes from the GOBP Regulation of Syncytium Formation by Plasma Membrane Fusion, Osteoclast Fusion, and Nucleus Organization gene sets. While general patterns of enrichment could be observed in OC samples for the Syncytium Formation gene set for four donors (Fig. 5B), expression patterns for specific genes were more variable for Pairs 2, 3 and 6 (Supplementary Fig. 2B). Similar variation emerged for the other two gene sets (Supplementary Fig. 3A, B).

Finally, our data highlights the translational value of a purified human osteoclast dataset. Although murine models have advanced important discoveries in the context of bone biology, species-specific differences do exist in the bone and bone marrow microenvironment[43,44]. Specifically, human immune and stromal components are lacking or insufficient, even in humanized mice[45–47]. For example, preclinical ZA and denosumab studies performed in a xenograft PC model with humanized cancer-associated fibroblasts, lymphatic and blood endothelial cells, and humanized tissue-engineered bone constructs (hTEBC), demonstrated response to ZA but not to denosumab, a human-specific RANKL inhibitor[46,47]. In clinical trials, however, denosumab outperformed ZA in overall survival and delayed onset of skeletal-related events in multiple solid cancers, suggesting the absence of human osteoclasts limits the translational value of humanized mouse models in pre-clinical screening[48,49].

Genes regulating osteoclast formation and biology may also differ between species. Strawberry notch homologue 2 (*SBNO2*), for example, in the Osteoclast Fusion gene set, was demonstrated to regulate osteoclast fusion through a *DCSTAMP* mechanism in mouse models[50]. Our data, however, showed very little expression of *SBNO2* in our osteoclast samples, despite high expression of other fusion genes, suggesting the need for further investigation to validate the role of *SBNO2* and of other OC biomarkers in fully humanized bone models (Fig. 5C).

In conclusion, our live cell sorting protocol allows the isolation of mature, viable and functional primary human OC and creation of RNAseq datasets representing purified mature human OC to establish OC specific gene expression profiles. Furthermore, this study has built a foundation for a robust dataset for human OCs with matched $CD14^+$ precursor monocytes, unsorted and sorted mononucleated OCs to allow better understanding of changes in OC gene expression patterns that associate with unique maturation phases in OC differentiation and their contribution to composition of bone networks. To our knowledge, in this study we report the first transcriptomic gene expression profile dataset derived from purified primary human osteoclasts. We expect that the signatures generated in this study will aid deconvolution of heterogenous bone microenvironments such as tissue biopsy specimens and support development of translationally relevant model system to study bone biology.

In future directions, transcriptomic analysis of purified, differentiated osteoclasts will be utilized to develop gene expression signature panels for studies of the bone microenvironment and investigation of sub-cultured sorted osteoclasts for mechanistic studies and drug development.

### Declarations

## DECLARATION OF INTERESTS

There are no competing financial interests in relation to the work described. The following is a list of the authors' total disclosures. D.J.B. holds equity in Bellbrook Labs LLC, Tasso Inc., Salus Discovery LLC, Lynx Biosciences Inc., Stacks to the Future LLC, Flambeau Diagnostics LLC, Navitro Biosciences, and Onexio Biosystems LLC. D.J.B. is also a consultant for Abbott Laboratories. K.T.H has a family member who is an employee of Epic Systems. M.B has a family member who is an employee of Luminex. S.G.Z reports unrelated patents licensed to Veracyte, and that a family member is an employee of Artera and holds stock in Exact Sciences. J.M.L has consulted for Pfizer, Janssen, Macrogenics, Foundation Medicine, Gilead, Arvinas, Astellas, Cytogen, and Seattle Genetics.

## AUTHOR CONTRIBUTIONS

A.B.D, E.H. designed the experiments and wrote the draft. A.B.D., E.H., N.S., C.S.D. developed methodology. A.B.D., E.H., S.R.R., X.T.H. carried out experiments and analyzed the data. A.B.D., E.H., M.L.B., S.G.Z. contributed to data analyses. All authors revised the manuscript and provided feedback. J.M.L. and D.J.B conceived the study, secured funding and provided oversight for the research.

## ACKNOWLEDGMENTS

We thank Dr. Jamie Sperger, Charlotte Linebarger, Leilani Mora Rodriguez for their support and expert advice with cDNA and library preparations for sequencing. This work was supported by NIH

UG3CA260692, NIH P50CA269011, Prostate Cancer Foundation Tactical Award 23TACT01, the University of Wisconsin, Carbone Cancer Center Support Grant NIH P30CA014520, Medical Scientist Training Program Grant T32 GM140935, The Veterans Affairs Advanced Fellowship in Women's Health. Figures were created with BioRender.com and Adobe Illustrator.

## DATA AVAILABILITY

The experimental data is available under request to the corresponding author.

## Tables

#### Table 1. List of Reagents

| Protocol | Company | Component | Catalog # | RRID # |
|---|---|---|---|---|
| **Blood processing** | Miltenyi | CD14 Microbeads | 130-050-201 | AB_2665482 |
| | Miltenyi | LS Columns | 130-042-401 | |
| | BD | BD Vacutainer EDTA Tubes | 367863 | |
| | Corning, Thermo Fisher Scientific | Hank's Balanced Salt Solutions | 21022CM | |
| | Cytiva, Thermo Fisher Scientific | Cytiva Ficoll-Paque PLUS Media | 45001750 | |
| **Osteoclast Culture and Differentiation** | | | | |
| | | | | |
| | | | | |
| Macrophage Differentiation Media | Corning, Thermo Fisher Scentific | Corning™ DMEM With L-Glutamine and 4.5g/L Glucose; Without Sodium Pyruvate | MT10017CV | |
| | R&D Systems | Fetal Bovine Serum | S11550H | |
| | DOT Scientific | Penicillin/Streptomycin | DSP93560-50 | |
| | R&D Systems | Macrophage Colony Stimulating Factor | 216-MC-025/CF | |
| | | | | |
| Osteoclast Differentiation Media | Corning | Dulbecco's Modified Eagle Medium | MT10017CV | |
| | R&D Systems | Fetal Bovine Serum | S11550H | |
| | DOT Scientific | Penicillin/Streptomycin | DSP93560-50 | |
| | R&D Systems | Macrophage Colony Stimulating Factor | 216-MC-025/CF | |
| | PeproTech | RANK-L | 310-01 | |
| **Osteoclast Sort** | | | | |
| | Innovative Cell Technologies, | Innovative Cell Technologies ACCUTASE | NC9839010 | |

| | | | | |
|---|---|---|---|---|
| | Thermo Fisher Scientific | CELL DETACHMENT REAGN | | |
| | ThermoFisher | UltraComp eBeads Compensation Beads | 01-2222-42 | |
| Flow Cytometry Antibodies | Invitrogen, ThermoFisher | RANK Monoclonal Antibody (9A725), PE | MA1-41015 | AB_1087023 |
| | BD Pharmingen | FITC Mouse Anti-Human CD51/61, Clone 23C6 (RUO) | 555505 | AB_2129630 |
| | Biolegend | Alexa Fluor® 647 anti-human CD14 Antibody | 325612 | AB_830685 |
| | | | | |
| Dyes | ThermoFisher | Hoechst 33342 solution, 20 mM | 62249 | |
| | Cytek Tonbo | Ghost Dye 780 | 13-0865-T100 | |
| | | | | |
| **RNA extraction** | Qiagen | Qiashredders (250) | 79656 | |
| | Qiagen | RNAeasy Plus Micro Kit (50) | 74034 | |
| | | | | |
| **cDNA Synthesis** | Takara | SMART-Seq mRNA Kit | 634773 | |
| | Illumina | Nextera XT Library Prep Kit | 15032354 | |
| | | IDT DNA/RNA UD Indexes Set A | 20027213 | |
| **Library Preparation** | | IDT DNA/RNA UD Indexes Set B | 20027214 | |
| | | IDT DNA/RNA UD Indexes Set C | 20042666 | |
| | | IDT DNA/RNA UD Indexes Set D | 20042667 | |
| | | | | |

| | | | |
|---|---|---|---|
| **RNA/cDNA Quantification - Agilent TapeStation 4200** | Agilent Technologies | HighSensitiviy RNA ScreenTape | 5067-5579 |
| | | High Senstivity RNA Sample Buffer | 5067-5580 |
| | | High Sensitivity RNA Ladder | 5067-5581 |
| | | D1000 ScreenTape | 5067-5582 |
| | | D1000 Reagents | 5067-5583 |
| | | D5000 High Sensitivity ScreenTape | 5067-5592 |
| | | D5000 High Sensitivity Reagents | 5067-5593 |
| **RNA Quantification - Qubit** | Invitrogen | Qubit RNA HS Assay Kit | Q32855 |
| **qPCR** | | | |
| | | | |
| Reverse Transcription | Applied Biosystems, ThermoFisher | High-Capacity RNA-to-cDNA™ Kit | 4387406 |
| PreAmp master mix | Applied Biosystems, ThermoFisher | TaqMan™ PreAmp Master Mix (cDNA pre-amplification for qPCR) | 4391128 |
| iTaq probes | BioRad | iTaq Universal Probes Supermix | 1725134 |
| Taqman Primers | ThermoFisher | ACP5 Hs00356261_m1 | 4331182 |
| | | CTSK Hs00166156_m1 | 4331182 |
| | | MMP9 Hs00957562_m1 | 4331182 |
| | | CD163 Hs00174705_m1 | 4331182 |
| | | POLR2A Hs00172187_m1 | 4331182 |

## Figures

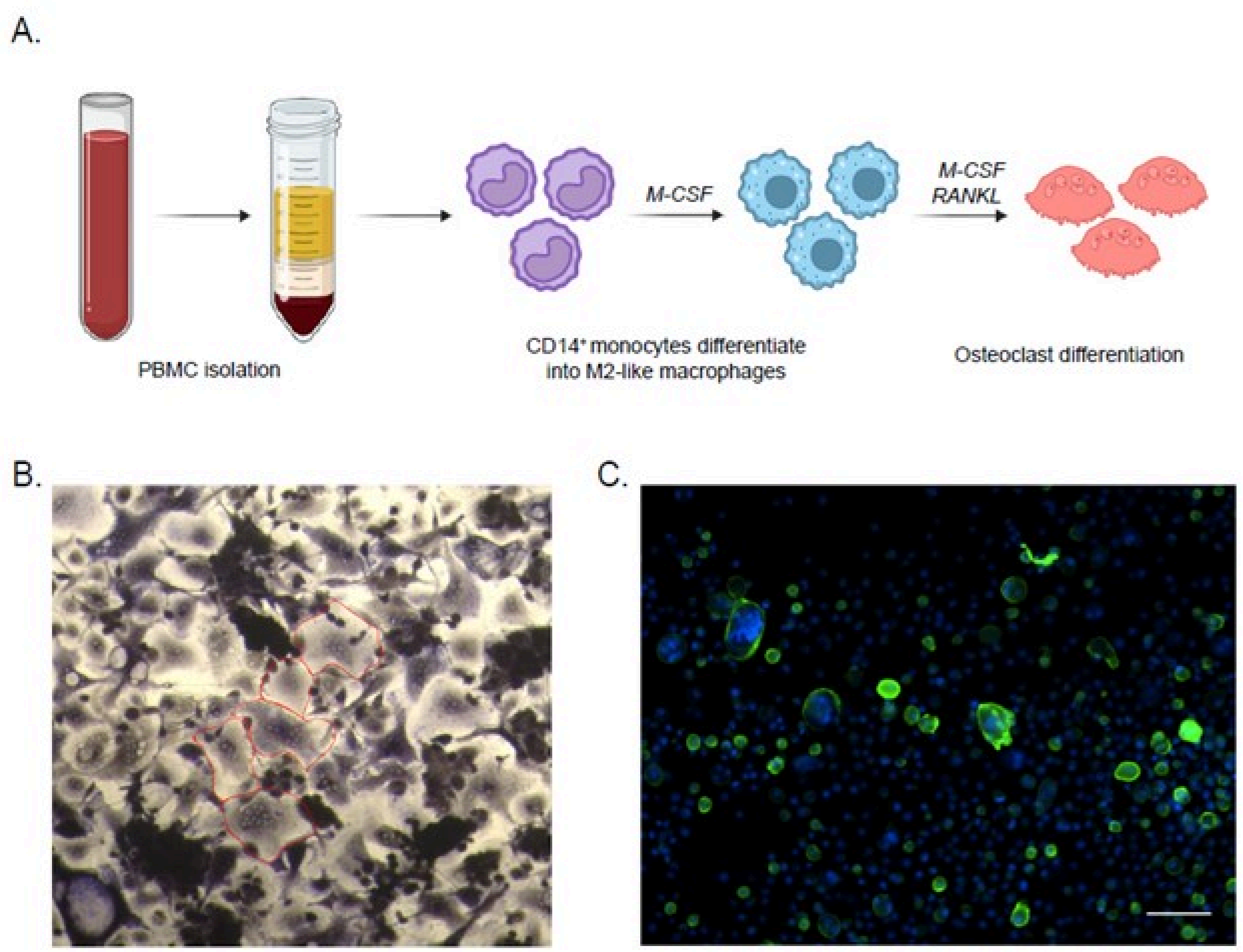

**Figure 1**

**Differentiation of monocyte-derived primary human osteoclasts. (a)** Schematic overview of osteoclast differentiation culture (b) Brightfield microscopy visualization of stained tartrate resistant acid phosphatase (TRAP) on differentiated osteoclast culture with osteoclasts (some representative osteoclasts outlined in red) (c) Fluorescent microscopy image of positive CD51/61 (green) binding on bulk osteoclast culture; nuclei stained with Hoechst (blue). Scale bars represent 100 mm.

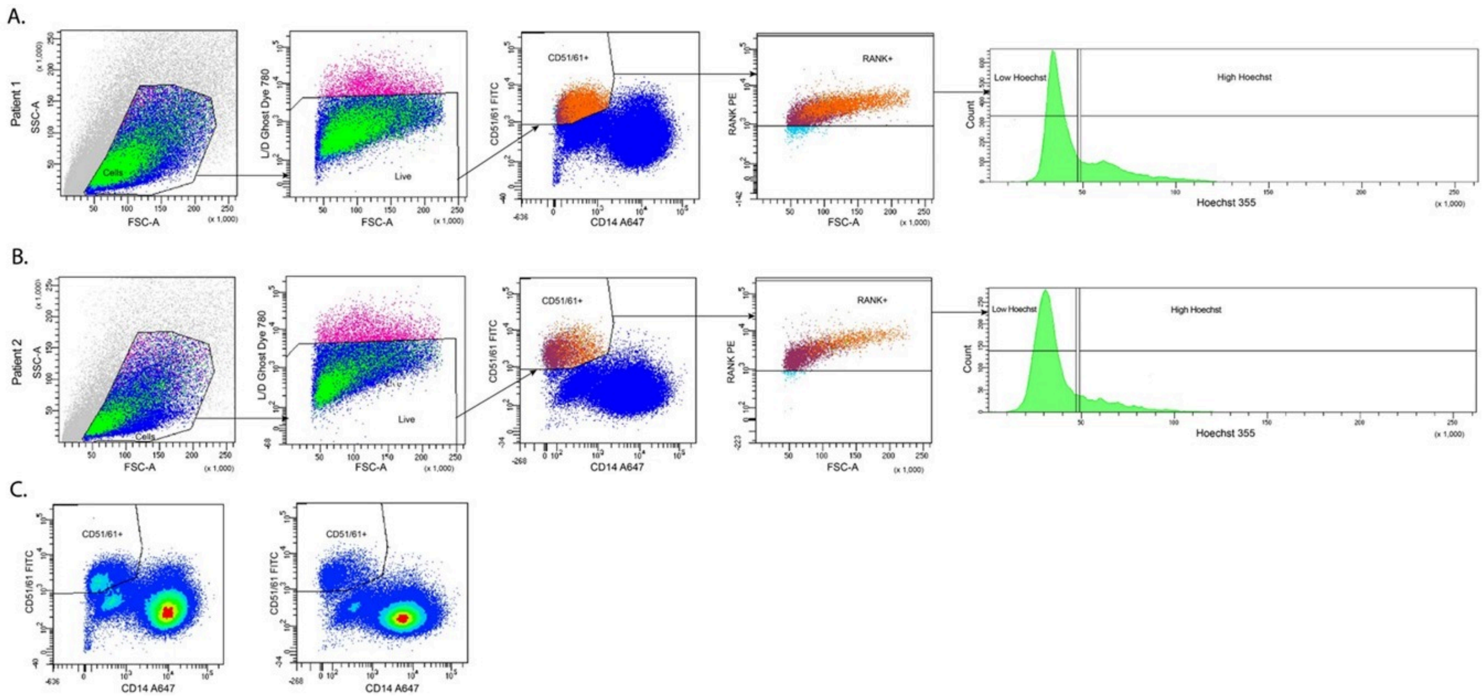

### Figure 2

**Live Cell Sorting strategy for monocyte-derived osteoclasts (a, b)** Representative Gating Strategy plots for two independent donors, Patient 1 and Patient 2. Live cells were gated on CD51/61 and CD14 expression. Next, $CD51/61^{+}CD14^{-}$ cells were projected on the RANK spectrum and $RANK^{+}$ cells were sorted into Low and High Hoechst populations **(c)** Density plots of CD51/61 vs CD14 binding shows gate for Patient 1 and Patient 2, demonstrating stratification of cells into three distinct subpopulations and representative gate placement to enrich $CD51/61^{+}$ $CD14^{-}$ cells.

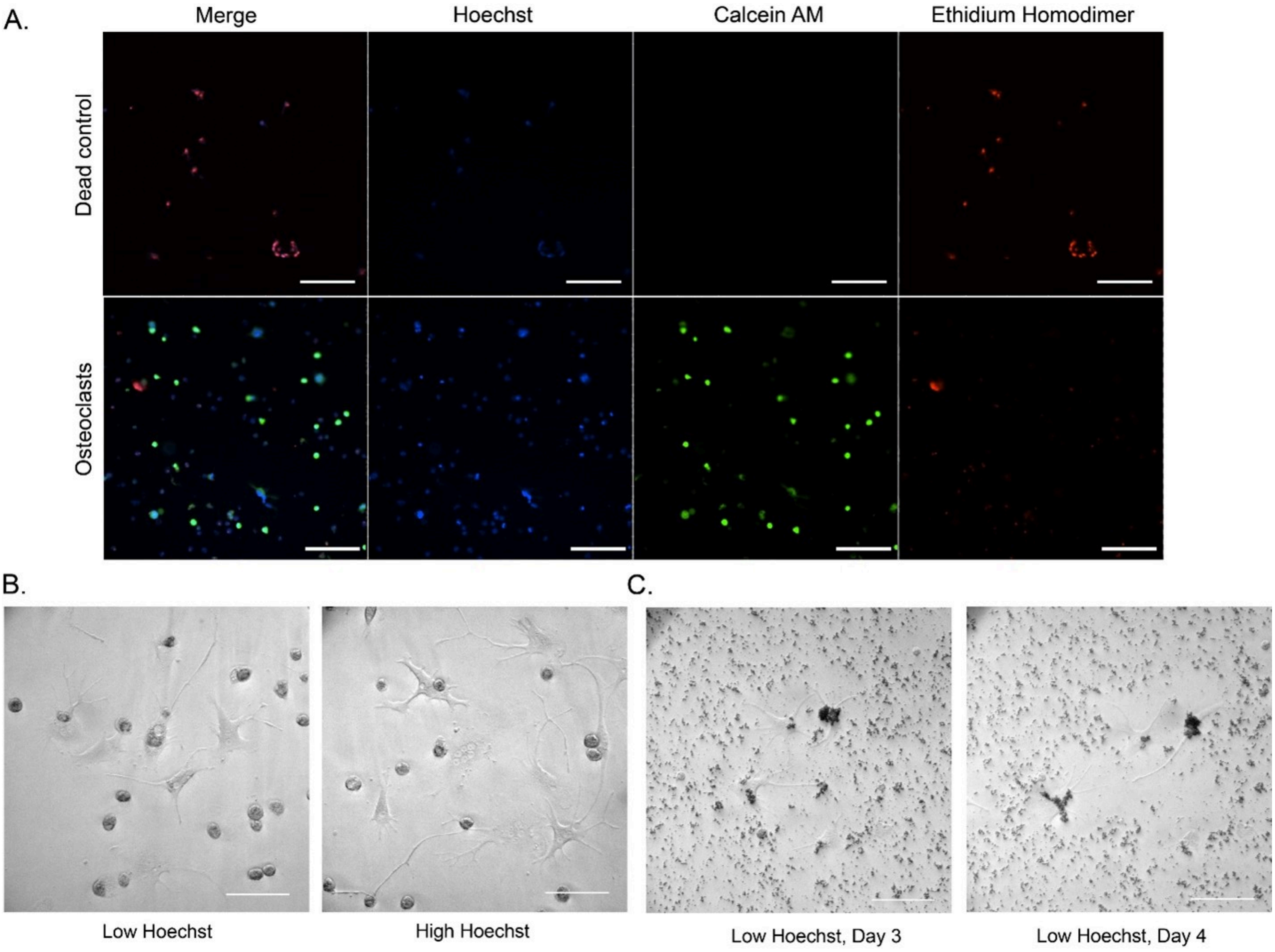

### Figure 3

**Evaluation of osteoclast viability and function after live cell sorting. (a)** Fluorescent microscopy images of Calcein AM (green)/ethidium homodimer (red) viability assay **(b)** Brightfield images of sorted ‘Low Hoechst’ and ‘High Hoechst’ cells at Day 3 in sub-culture following live cell sorting. Membrane extensions and multinucleated cells are visible after adhesion **(c)** Serial matched brightfield microscopy images demonstrate ferromagnetic bead uptake by sorted ‘Low Hoechst’ cells at day 3 and 4 after live cell sorting. Scale bar*s* represent 100 mm.

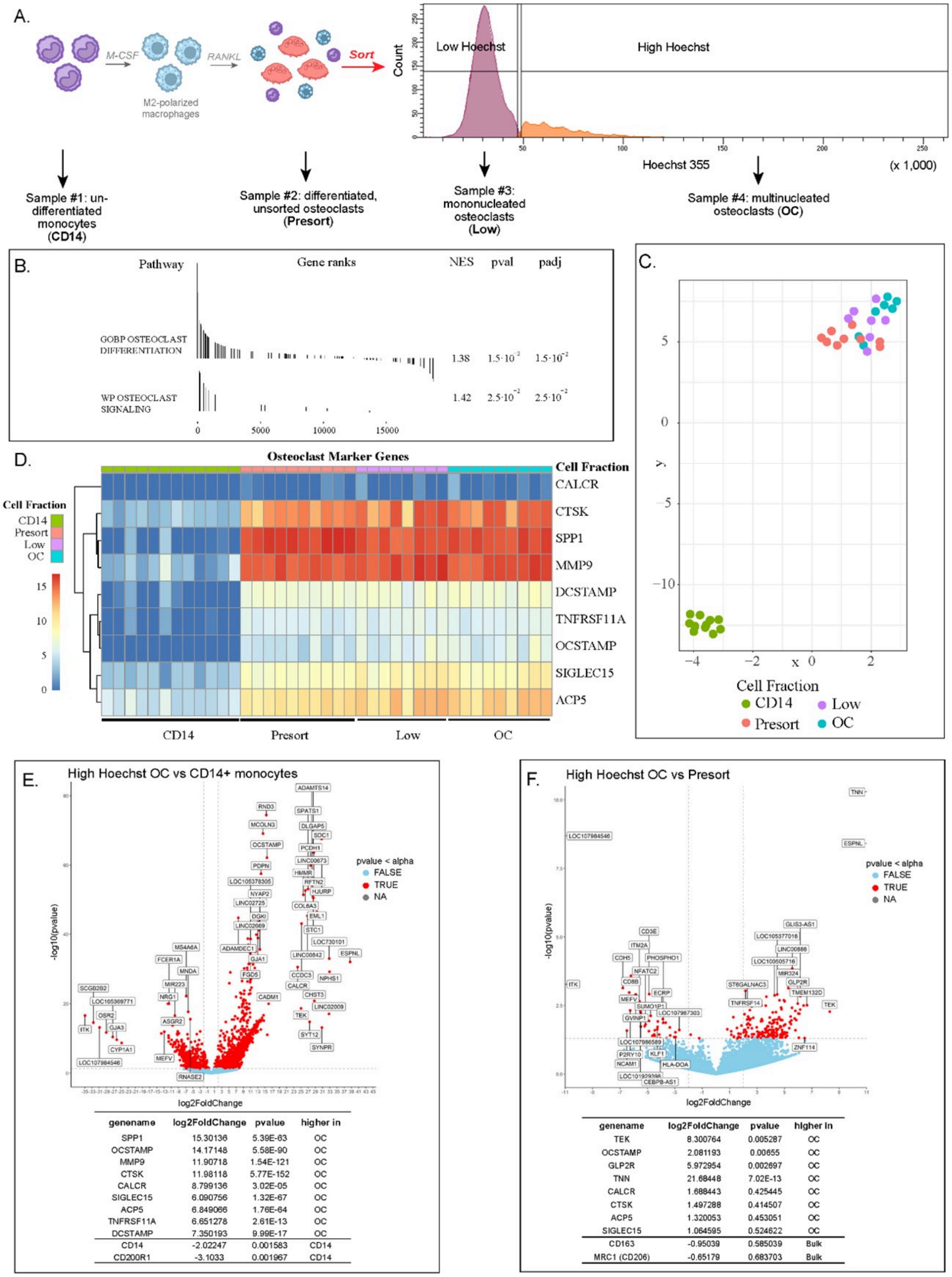

| genename | log2FoldChange | pvalue | higher in |
|---|---|---|---|
| SPP1 | 15.30136 | 5.39E-63 | OC |
| OCSTAMP | 14.17148 | 5.58E-90 | OC |
| MMP9 | 11.90718 | 1.54E-121 | OC |
| CTSK | 11.98118 | 5.77E-152 | OC |
| CALCR | 8.799136 | 3.02E-05 | OC |
| SIGLEC15 | 6.090756 | 1.32E-67 | OC |
| ACP5 | 6.849066 | 1.76E-64 | OC |
| TNFRSF11A | 6.651278 | 2.61E-13 | OC |
| DCSTAMP | 7.350193 | 9.99E-17 | OC |
| CD14 | -2.02247 | 0.001583 | CD14 |
| CD200R1 | -3.1033 | 0.001967 | CD14 |

| genename | log2FoldChange | pvalue | higher in |
|---|---|---|---|
| TEK | 8.300764 | 0.005287 | OC |
| OCSTAMP | 2.081193 | 0.00655 | OC |
| GLP2R | 5.972954 | 0.002697 | OC |
| TNN | 21.68448 | 7.02E-13 | OC |
| CALCR | 1.688443 | 0.425445 | OC |
| CTSK | 1.497288 | 0.414507 | OC |
| ACP5 | 1.320053 | 0.453051 | OC |
| SIGLEC15 | 1.064595 | 0.524622 | OC |
| CD163 | -0.95039 | 0.585039 | Bulk |
| MRC1 (CD206) | -0.65179 | 0.683703 | Bulk |

### Figure 4

**Bulk RNAseq Analysis of sorted and unsorted monocyte-derived osteoclast cells and CD14 monocytes.** (**a**) Schematic overview of experimental approach and analytes generated for RNA sequencing. **(b)** Gene set enrichment analysis of High Hoechst osteoclasts showing significant enrichment for GOBP Osteoclast Differentiation and WP Osteoclast Signaling gene sets **(c)** Uniform Manifold Approximation and Projection (UMAP) clustering of RNAseq samples shows distinct CD14, Low Hoechst (Low), bulk

(Presort), and High Hoechst osteoclast (OC) populations **(d)** Heatmap of log2(TPM+1) normalized gene expression of hallmark osteoclast genes **(e)** Volcano plot of gene expression change comparing sorted OC versus CD14$^{+}$ monocyte precursors **(f)** Volcano plot of gene expression change comparing OC versus Presort bulk osteoclast specimen.

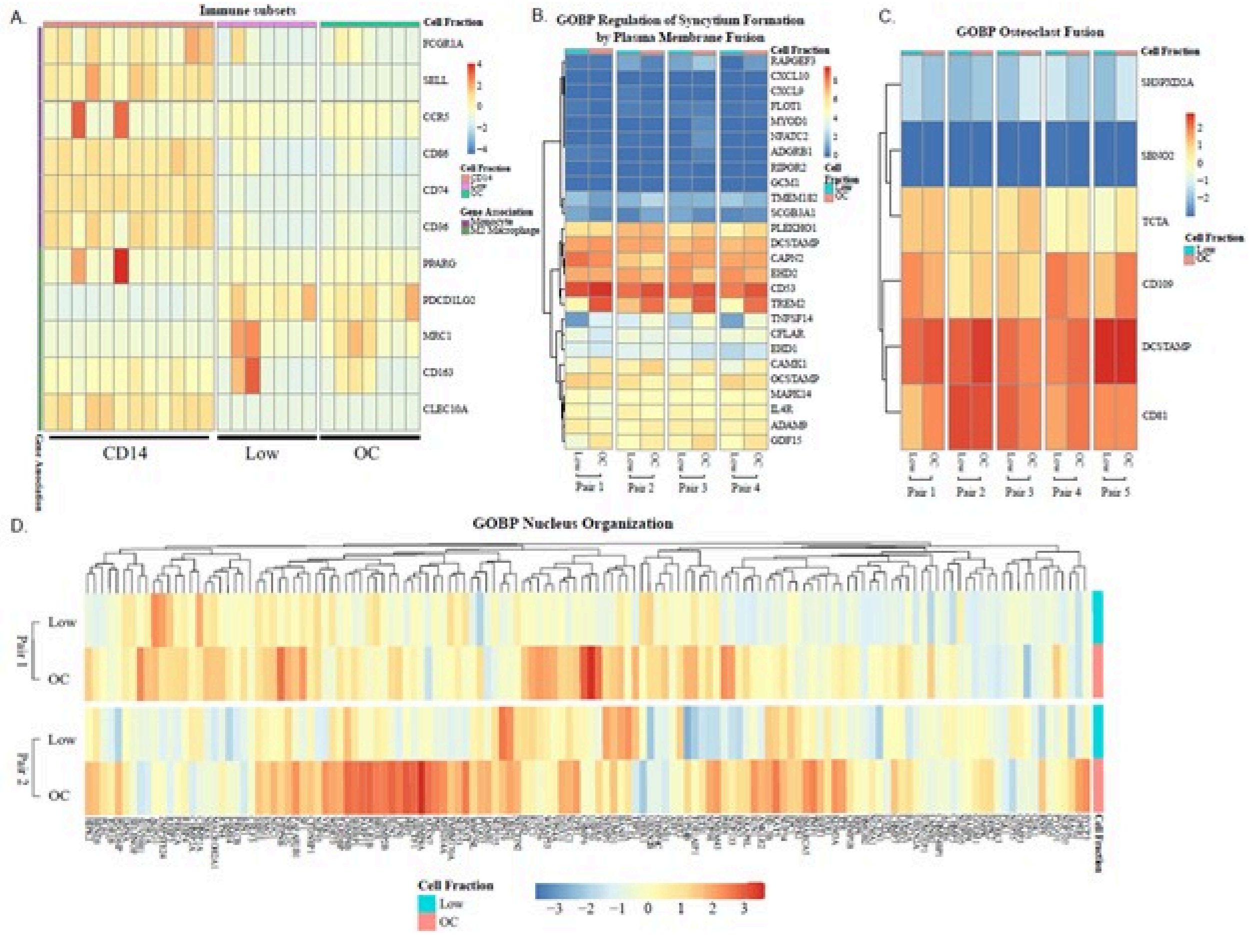

**Figure 5**

**Heatmaps of immune cell subsets and gene sets associated with biological processes (a)** Scaled gene expression for monocyte and M2 macrophage-associated genes, comparing CD14$^{+}$ monocytes, Low, and OC sorted osteoclast subsets **(b)** Normalized gene expression for the GOBP Regulation of Syncytium Formation by Plasma Membrane Fusion gene set, comparing sorted Low and OC matched pairs **(c)** Normalized gene expression for the GOBP Osteoclast Fusion gene set, comparing sorted Low and OC matched pairs **(d)** Scale gene expression for the GOBP Nucleus Organization gene set for two matched Low and OC pairs.

## Supplementary Files

This is a list of supplementary files associated with this preprint. Click to download.

- Suppfigs.docx
